# Primary malignant melanoma of the parotid gland, a rare case arising from a duct: a case report and literature review

**DOI:** 10.3389/fonc.2024.1324214

**Published:** 2024-06-06

**Authors:** Moe Thu Zar Aung, Jo-Eun Kim, Hye-Jung Yoon, Kyoung-Hoe Huh, Won-Jin Yi, Min-Suk Heo, Sam-Sun Lee

**Affiliations:** ^1^ Department of Oral and Maxillofacial Radiology, School of Dentistry and Dental Research Institute, Seoul National University, Seoul, Republic of Korea; ^2^ Department of Oral Medicine, University of Dental Medicine, Mandalay, Myanmar; ^3^ Department of Oral Pathology, School of Dentistry and Dental Research Institute, Seoul National University, Seoul, Republic of Korea

**Keywords:** parotid gland, malignant melanoma, computed tomography, magnetic resonance imaging, diagnosis

## Abstract

Malignant melanoma of the parotid gland is an unusual tumor in the head and neck region, and most parotid melanoma is reported as a metastatic lesion of cutaneous malignant melanoma. We report a case of primary malignant melanoma arising in the parotid gland duct with diagnostic challenge. The patient was a 68-year-old man who complained of repeated right facial swelling that presented 3 months prior. Swelling was detected along the Stensen’s duct of the cheek, and brown-colored saliva-like fluid was aspirated. On MR and CT images, a fluid-filled duct with small nodule and heterogeneously enhancing mass in the parotid parenchyma was detected. The nodular mass on the ductal wall grew rapidly, and the hyperintense T1 signal became significant on follow-up images. The final diagnosis via histopathologic examination using biopsy and parotidectomy specimen revealed the lesion as malignant melanoma of the duct and pleomorphic adenoma of the parenchyma. Even if the incidence of primary malignant melanoma is very low among tumors occurring in the parotid gland, efforts supporting an early diagnosis using imaging characteristics are important.

## Introduction

Malignant melanoma (MM) is a malignant tumor of uncontrolled replication of pigment-producing melanocytes within the skin epidermis. Cutaneous melanomas are the most common type of melanoma in the head and neck region (85%~90%), whereas mucosal melanoma also can arise in the oral cavity (1). MM rarely affects the parotid gland and presents as a progressively enlarging, asymptomatic, firm, and fixed parotid mass. These parotid melanomas are assumed to originate from intraparotid lymph node metastasis, which primarily arises from skin neoplasm of the head and neck (2–4).

The origin of primary MM can be difficult to determine or may never be identified (5), indicating the possibility of primary MM of the parotid gland. In addition, the presence of melanocytes in the normal parotid gland supports the possibility of primary MM of parotid glands (6, 7). Although rare, some studies reported primary MMs of the parotid gland characterized by poor prognosis and difficult delayed diagnosis (8–10). However, there has been no report of MM in the parotid duct until now.

In general, clinical findings such as pigmentation could act as a pathognomonic finding when diagnosing cutaneous melanoma. In the case of parotid MM, which does not have the crucial clinical sign of pigmentation, help with radiologic or pathologic examination is urgently needed. Diagnostic imaging is useful for identifying MM because melanoma shows hyperintense signal intensity on T1-weighted images due to the presence of melanin (11).

The following case describes a primary MM thought to have originated from the parotid gland, especially the duct, and that involved diagnostic difficulties due to an incidentally concurrent parenchymal parotid mass. In this case report, CT and MRI features of primary MM will be reviewed throughout the literature.

## Case presentation

A 68-year-old male patient visited the neurosurgery (NS) department with repeated right facial swelling along the Stensen’s duct that first presented 3 months prior. The patient had no other specific underlying disease. Before visiting this hospital, he had undergone several procedures to drain the fluid from the duct in the local clinic, but the swelling returned each time. There were no neurological abnormalities, and NS surgeon referred the patient to the department of oral and maxillofacial surgery (OMFS) under the clinical diagnosis of obstructive sialadenitis due to sialolith. Salivation via a duct orifice was confirmed upon massaging the parotid gland, and aspiration confirmed a brown-strawberry–colored, non-mucous fluid with hemosiderin precipitate. The OMFS surgeon diagnosed the mass as a parotid duct cyst with vascular malformation and phlebolith.

**Figure 1:**
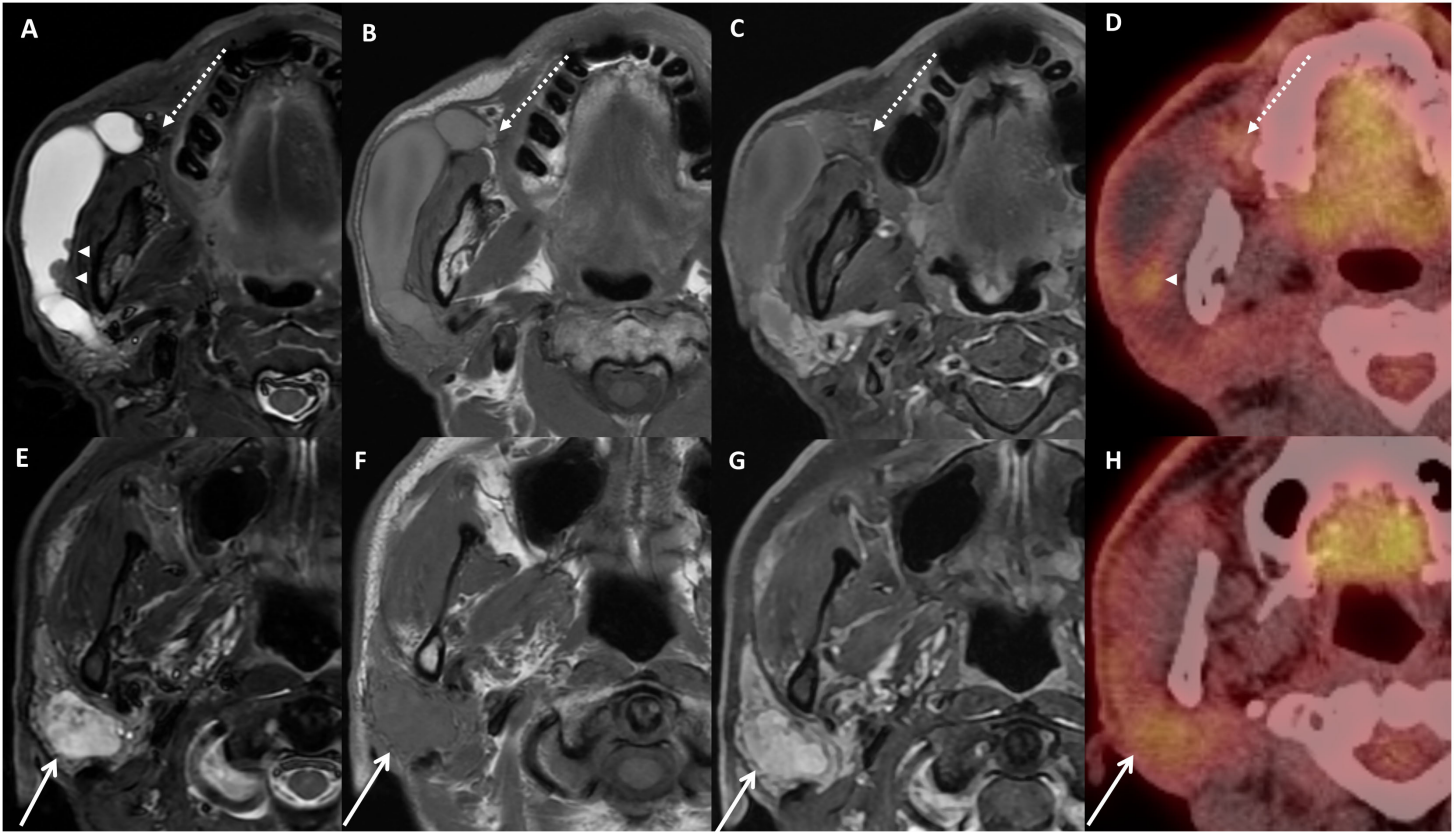
Images of initial imaging examinations. Axial MRI with fat-suppressed T2-weighted image **(A, E)**, T1-weighted image **(B, F)** fat-suppressed contrast-enhanced T1-image **(C, G)**, and PET-CT images **(D, H)**. Dilated duct filled with fluid signal, and small nodules on the ductal wall show slight hyperuptake on PET image **(A**, **D**, arrowhead**)**. Note the small focus at the orifice region with hypointense T2 and hyperintense T1 signal **(A–C**, dotted arrow**)**. A heterogeneous mass with enhancement on contrast-enhanced MRI **(G)** and slight hyperuptake on PET **(H)** in the parenchyma were detected **(E–H**, arrow**)**. Nodules on ductal wall and parenchymal mass showed different signal entity.

In the initial MR image, prompt dilation filled with hyperintense T2, non-enhancing signal of right Stensen’s duct was confirmed, and a hypointense nodular focus was observed in the duct orifice region (**Figures 1A–D**). Some small nodular foci were detected in the dilated ductal wall, and these nodules showed hypointense T2 and enhancement signals. In addition, in the parotid parenchyma, an enhancing mass was found, and the mass showed heterogeneous hyperintense T2 and hypointense T1 signal intensity (**Figures 1E–H**). The low-signal nodule at the orifice was thought to be sialolith causing obstruction and sialadeitis in the NS (**Figures 1A, B**). However, it also could be regarded as phlebolith combined with venolymphatic malformation, considering the parenchymal lesion as part of the same lesion. PET-CT images showed minimal hyper-uptake in the parotid parenchyma, hindering a malignancy diagnosis (**Figure 1H**).

**Figure 2:**
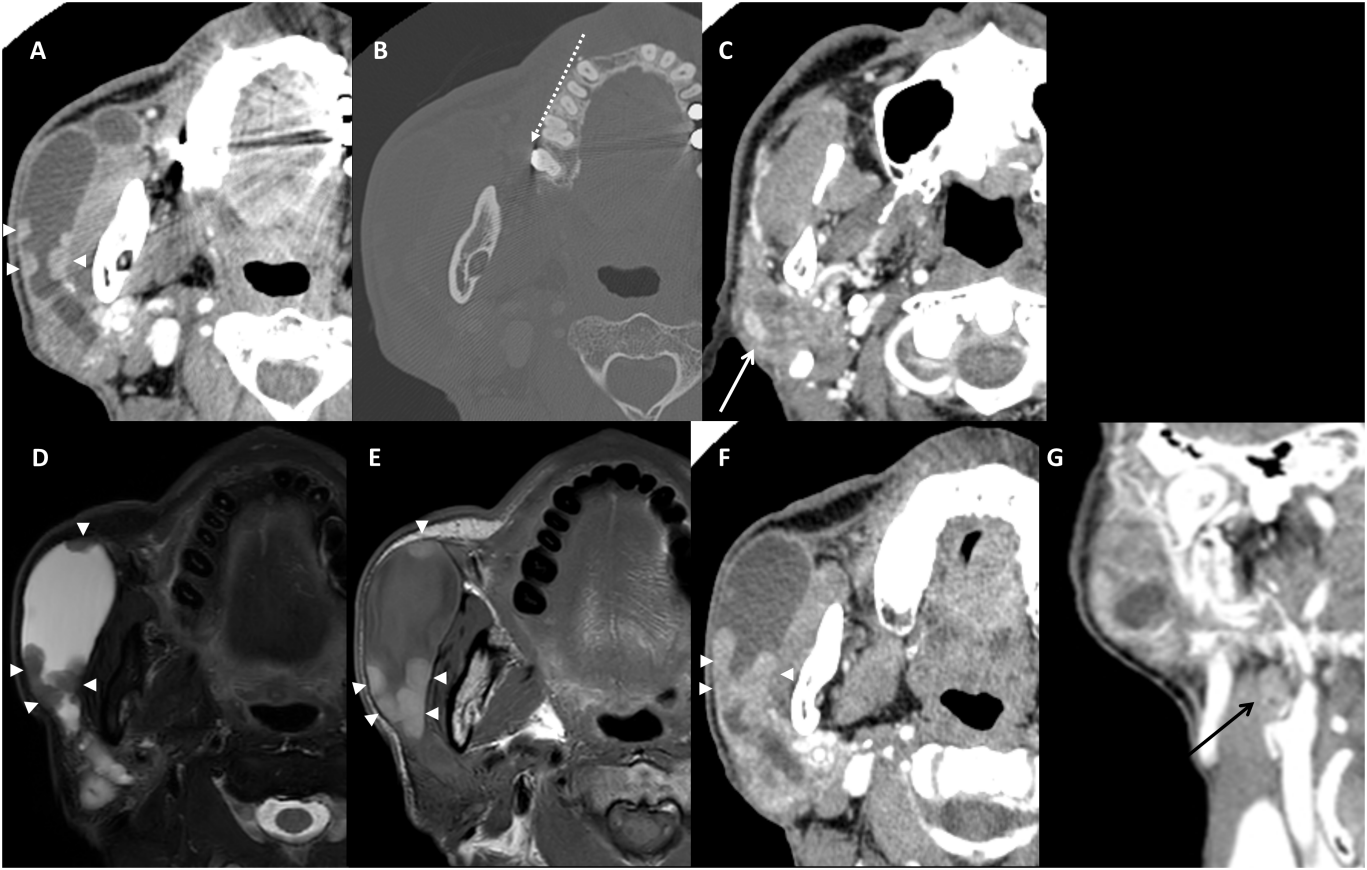
Contrast-enhanced CT images obtained after 1 month **(A–C)**. Nodular enhancing masses along the dilated ductal wall were enlarged **(A)**. No calcified focus at the orifice region on bone filtered reconstruction **(B)** dotted arrow. Parenchymal lesion showed heterogeneous enhancement **(C)** white arrow. Pre-operative images (the images 3 months after the initial MR) of axial fat-suppressed T2-weighted MR image **(D)**, T1-weighted MR image **(E)**, contrast-enhanced CT images of axial **(F)**, and coronal **(G)**. An enlarged ductal wall and enlarged nodular mass (arrowheads) were confirmed **(D–F)**. Note the hypointense T2 signal and hyperintense T1 signal of ductal wall melanoma **(D, E)**. Coronal contrast-enhanced CT image shows an enlarged ipsilateral cervical lymph node that is revealed as metastasis (black arrow, **(G)**.

In the contrast-enhanced CT images obtained after a month, nodular enhancing masses along the dilated ductal wall were confirmed (**Figure 2A**), but there was no calcified focus at the orifice (**Figure 2B**). The heterogeneously enhancing parenchymal mass showed a mixture of highly attenuated areas similar to vessel and a low attenuated lesion with faint margin (**Figure 2C**). Considering the MR/CT images and clinical findings, the clinical diagnosis of OMFS surgeon was venolymphatic malformation.

Intra-oral open biopsy via the orifice region of the buccal cheek mucosa was performed. When the incision and slight dissection were performed, a black tumor nodule was revealed and sent to the oral pathology department. The OMFS surgeon confirmed no pigmentation in the intraoral mucosa near the orifice. Histopathologic examination confirmed the biopsy specimen as MM.

Screening of other sites especially the head and neck skin was performed but showed no pigmented lesion. On pre-operative CT and MR images obtained 3 months after the initial imaging, the nodular lesion on the ductal wall was enlarged (**Figure 2D**), the hyperintense T1 signal (**Figure 2E**) and enhancement (**Figure 2F**) became remarkable. In addition, a positive lymph node not detected in the previous exams was enlarged to level IIa (**Figure 2G**). However, the parenchymal mass showed no change in size or characteristics.

**Figure 3:**
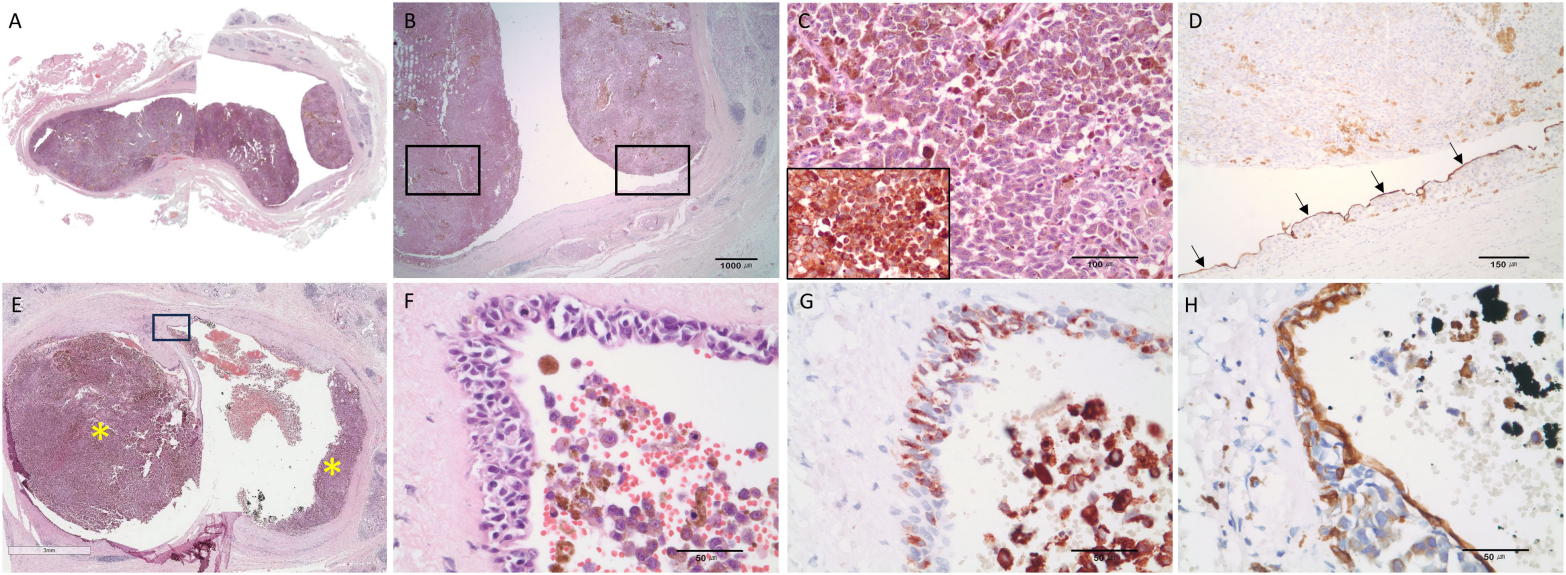
Representative histopathologic features strongly suggesting that malignant melanoma originated from the ductal epithelium. **(A, B)** Malignant melanoma of the parotid duct. Intraluminal masses arising from the ductal epithelium. **(C)** Large polygonal tumor cells had melanin pigments and immunoreactivities for HMB-45. **(D)** The remaining ductal epithelial cells were detected by the immunostaining with cytokeratin 7 (black arrows). **(E)** Tumor masses (asterisk) arising from the ductal epithelium (scale bar = 3mm). Inserted box area is magnified and shown in **(F–H)**. **(F)** Hyperchromatic, atypical cells were observed in the ductal epithelium. **(G)** Proliferating atypical melanocytic cells in the epithelium were positive for HMB-45. **(H)** The intact ductal epithelial cells were positive for cytokeratin 7. Hematoxylin and eosin staining **(A–C, E, F)** and immunohistochemical staining **(D, G, H)**; Olympus BX45 microscope; Spot image capture software (Spot Imaging, Sterling Heights, MI, USA). HMB45.

The operation including Mass excision with total parotidectomy, selective neck dissection, latissimus dorsi muscle free flap, and facial nerve repair with sural nerve graft was performed. In the specimen sent to the oral pathology department, two different lesions in the parotid gland were confirmed histopathologically. One was a tumor mass that had grown into the lumen from the epithelium of an enlarged salivary gland duct (**Figures 3A, B, E**). The tumor cells had dark brown melanin pigments in hematoxylin-eosin staining (**Figure 3F**) and showed diffuse positive immunoreactivity for HMB-45 (**Figures 3C, G**). The remaining ductal epithelial cells of the affected duct were positive for cytokeratin 7 (**Figures 3D, H**). The other mass is a pleomorphic adenoma that existed in the deeper part of the parotid gland. Therefore, the final diagnosis was MM of the salivary duct accompanied by pleomorphic adenoma. Ipsilateral lymph node metastasis of MM was confirmed at levels Ib and IIa.

Post-operative radiation therapy and chemotherapy were performed as adjuvant treatments. The rehabilitation treatment for facial paralysis continues. Although the patient complained of reduced function in trigeminal and facial nerves (altered sensation and impaired eyebrow movement), there was no evidence of local recurrence on follow-up CT, MR, or PET images up to 13 months after the operation.

## Discussion

Tumors of the salivary glands are rare, accounting for less than 3% of all head and neck neoplasms. Off all salivary gland tumors, 80% develop in the parotid gland and 25% of those are malignant (12). The parotid gland itself can act as the filtering station for lymphatic drainage in the head and neck. Therefore, malignancy of the head and neck region most commonly leads to metastasis to the parotid gland. A previous study reported that about 80% of parotid metastasis are assumed to originate from a cutaneous malignancy of the upper face and scalp (13).

MM of the parotid gland is very rare, and there remains controversy regarding its origin. Generally, MM is thought to form through lymph node drainage from cutaneous melanoma of head and neck primary cancers. However, the primary tumor source can be difficult to determine. About 10% to 35% of melanomas show regression and resolve completely. Some studies suggest that regressed MMs are more likely to metastasize (14). The majority of previously reported cases of primary parotid MM appears to represent metastasis within the intraparotid lymph node. Regression of primary cutaneous melanoma can occur through autoimmune surveillance.

However, many studies claim that melanocytes originally exist in a gland, which can give rise to MM with a primary origin. In a prior study, Takeda found melanocytes in the interlobular duct of the parotid gland during an autopsy on a Japanese male; melanocytes derive embryologically from the neural crest and do not usually form part of the salivary tissue (6). On the other hand, Greene and Bernier reported that the parotid gland may also contain melanoblasts because it develops from invaginating buccal epithelium that could contain melanoblasts and pointed out the presence of melanin within ductal and acinar cells. They also described primary MM as infiltrative, resulting in poorly demarcated tumors and not located in lymph nodes (7).

To diagnose primary MM of the parotid gland, the following criteria proposed by Woodward et al. should be met (15):

[1] The tumor mass is located within the parotid gland.
[2] The tumor does not contain any identifiable lymph node tissue.
[3] There is no evidence of other MM lesions in the body.
[4] There is no evidence of previous MM excision or progression of suspicious pigmented lesion.

The present case satisfies these four criteria and, unlike other cases, showed characteristic features above within a duct. Metastatic MM should involve the parenchymal lymph node but not the duct, supporting diagnosis of this lesion as primary MM of the parotid gland. Moreover, there was another primary mass (revealed as pleomorphic adenoma) in the parenchyma, hindering diagnosis using CT and MR images. Considering the entire diagnostic process, the MM of this case was thought to have arisen from the parotid ductal tissue and grown in the inner space of the main duct. It is possible that the mass originated from the oral mucosal tissue of the orifice and grew retrograde into the duct, although this possibility is low because the surgeon confirmed no pigmentation in the oral mucosa, including the orifice region. MM arising from a parotid duct has not been previously reported.

We reviewed papers in PubMed and Google Scholar using the keyword “parotid malignant melanoma.” Among the papers published since 1960, only English articles were reviewed, and cases of metastasis from an unknown primary site to the parotid LN were excluded. **Table 1** summarizes the clinical and imaging features from the documents.

**Table 1:** Summary of literature review of primary malignant melanoma of the parotid gland.

Publication year	Age/sex	Clinical presentation	Size (cm)	Metastasis to other site	Facial n. function	Imaging modality	Imaging features (margin)	Imaging features (internal)	Histopathologic exam	Other sites’ exam	Treatment	Prognosis (FU)	Note (initial diagnosis)
2020 (10)	27/F	Parotid swelling (8 mo)	5		Intact	MR	Moderate	T1 iso- and T2 hyperintense	Melanin A (+) and HMB-45 (+)	PET, pan endoscopy, and physical and imaging exams	P + RT + FR	DOD (11 mo)	
2018 (16)	13/M	Parotid swelling (6 mo), rapidly increased in size	12	CLN	Intact	CECT	Lobulating with skin invasion	Heterogeneously enhancing with necrotic foci	Melanin A (+), Vimentin (+), S100 (+), and HMB-45 (−)	Laryngoscopy and external exam	P (wide local excision) + SND	DOD (7 mo)	
2014 (17)	50/M	Painless mass of mandibular angle (3 mo)	3.8		Intact	US and MR	Well-defined, slightly lobulated (nodular)	US: heterogeneous with very little vascularization MR: heterogeneous, T1 high on MR	Melanin A (+), S100 (+), and HMB-45 (+)	Pan endoscopy, cranial/brain MR and CT, and physical and imaging exams	P with sural n. graft	NED (6 mo)	Initial diagnosis using US-guided FNAC was inconclusive
2008 (9)	37/M	Swelling (2 mo)	5	Multiple, CLN	Intact	CT	Obscure		Amelnotic malignant melanoma cells, S-100 (+), and HMB-45 (+)	Physical exam, GI endoscopy, abdominal US, and epipharyngoscopy	P	DOD (5 mo)	**Amelanotic malignant melanoma
2002 (8)	64/F	Parotid swelling (18 mo)	3.5	Multiple, CLN	Intact	CT			Neoplastic cells present variable amounts of melanin	Physical exam, immunoscintigraphy, and total-body CT	P + RND	DOD (9 mo)	
1999 (18)	60/F	Neoplastic enlargement (60 mo)			Intact	CT and MR	Dislocation of the major cervical v. by mass		S-100 (+) and HMB-45 (+)	Abdomen US, CT of thorax and brain, panedoscopy, and botal body scintiscan	P + RND	NED (60 mo)	Treated with RT for parotid ca. confirmed by FNAC
1993 (15)	51/F	Painless swelling of mandibular angle (9 mo)	4	CLN, IC	Intact	CT	Well-defined	Homogeneous	Melanin-containing malignant cells, S-100 (+), PGP9.5 (+)	Subsequent clinical exams and abdominal US	P + RND +CTx	DOD (15 mo)	
1990 (19)	28/F	Parotid swelling (6 mo)	6		Intact	CT		Homogeneous	S-100 (+), Fontana-Masson (+)		P + BCG immunotherapy	NED (48 mo)	
1961 (7)	23/M	(r/o) parotitis		Multiple organ	Unknown	n/a				Autopsy findings only	P	DOD (2 mo)	
	67/M	Parotid mass (3 yr), rapidly increase in size	4.5	IC	Unknown	n/a			Fontana's stain, Lillie's ferrous ion	General physical exam	E	DOD (12 mo)	
	28/M	Recurrent Parotid mass (initial Dx of adenoca)		Multiple	Unknown	n/a			Fontana's stain, Lillie's ferrous ion, melanin bleach	General physical exam	P	DOD (42 mo)	Undifferentiated adenocarcinoma
	41/M	Parotid mass			Unknown	n/a			Fontana's stain, Lillie's ferrous ion		P	DOD (48 mo)	Initial diagnosis from partial parotidectomy was inflamed hyperlpasia, however 2 month later, the diagnosis changed as undifferentiated epithelial tumor using FNAC.
	66/M	Parotid mass (2 mo)		CLN	Unknown	n/a			Fontana's stain, Lillie's ferrous ion, melanin bleach	Careful exams	P	NED (48 mo)	

FU, follow-up; F, female; M, male; MR, magnetic resonance; CT, computed tomography; CECT, contrast-enhanced computed tomography; PET, positron emission tomography; GI, gastrointestinal; P, parotidectomy; E, excision; RT, radiation therapy; FR, facial nerve rehabilitation; CLN, cervical lymph node; IC, intracranial; CTx, chemotherapy; RND, radical neck dissection; SND, selective neck dissection; BCG, Bacillus Calmette–Guérin; DOD, died of disease; NED, no evidence of disease; FNAC, fine-needle aspiration cytology; n/a, not applicable; (+), positive reaction; **, final diagnosis.

Most of the patients had non-specific symptoms such as parotid swelling, and the lesion size was large (3.5 cm to 12 cm) considering the symptom duration. All tumors occurred in the parenchyma and were located in some parts of the superficial lobe, or they replaced the entire parotid. However, there was no case located in the duct. Immunohistochemical staining, such as S-100 and HMB-45, was additionally performed for accurate diagnosis, and the possibility of melanoma in other regions was evaluated and excluded to prove primary MM.

In cases having images or description of images, the margins were relatively well-defined, and the internal view showed homogeneous or heterogeneous attenuation on CT imaging. Therefore, most of them were not clearly distinguished from benign salivary gland tumors such as pleomorphic adenoma, making it difficult to detect MM before biopsy. Although the patient number is small, hints of diagnosis were obtained from iso to high T1 signal in cases where MR was performed (10, 17), including this case. Hyperintense T1 signal in the MM indicated the paramagnetic effect of melanin and its accompanying hemorrhage (20). In this way, when diagnosing salivary gland tumor, MRI can not only provide information about the relationship between tumor and facial nerve for surgery but also contribute greatly to the diagnosis of rare diseases such as MM, so MRI is strongly recommended for diagnosing salivary gland tumor.

The treatments for primary MM of parotid gland were based on resection of the lesion, such as parotidectomy, and, sometimes, radiation therapy, chemotherapy, or immunotherapy were added. Metastasis to other sites was identified in 67% (8/12) of cases, except for follow-up loss cases and patients without metastasis having better prognosis [more NED (no evidence of disease) than DOD (died of disease), and longer follow-up (FU) duration]. In this review, most patients died of disease in the period of 2 to 48 months. We only reviewed the cases that insisted “primary” MM of parotid gland. According to previous studies, the prognosis of unknown primary parotid MM was better than known cutaneous MM, and the 5-year survival rate was more than 50% (21, 22). A full-scale comparative study on primary MM originating in the parotid gland and lesions metastasized to the parotid gland has not yet been conducted due to the small number of cases. However, based on this literature review, the two lesions may have different prognoses, so early exact diagnosis, more aggressive treatment methods, and closed follow-up may be needed for primary lesions.

Despite various arguments, there are also papers denying that primary MM originates in the parotid gland. In this case report, the absence of melanoma in other LNs or skin that could be assumed to be the primary lesion was not pathologically proven, so the possibility of occult skin melanoma in other areas cannot be completely ruled out. However, this case is very unique and has findings that suggest that it occurred within the duct, so it is worthy of reporting. Although the possibility of parotid primary MM is low, early diagnosis is very important. In the case of parotid mass, imaging exams were used for diagnosis and surgical planning. At this time, although it is very low, the possibility of MM should be not excluded even in the parotid duct, and image characteristics should be used for differential diagnosis. In particular, the hyperintense T1 signal shown in MR images can help diagnose MM as a key imaging feature.

## Data availability statement

The original contributions presented in the study are included in the article/supplementary material. Further inquiries can be directed to the corresponding author.

## Ethics statement

This study was approved by the Institutional Review Board of Seoul National University Dental Hospital (ERI230004). Written informed consent was obtained from the patient for the publication of any potentially identifiable images or data included in this article.

## Author contributions

MA: Conceptualization, Data curation, Methodology, Writing – original draft. J-EK: Data curation, Formal analysis, Investigation, Supervision, Validation, Writing – review & editing. H-JY: Formal analysis, Investigation, Validation, Writing – review & editing. K-HH: Conceptualization, Supervision, Writing – review & editing. W-JY: Supervision, Validation, Writing – review & editing. M-SH: Methodology, Supervision, Writing – review & editing. S-SL: Supervision, Validation, Writing – review & editing.
